# High Detection Frequency of Enteric Pathogens: Insight from Wastewater-Based Epidemiology (WBE) Surveillance Approach in Dakar, Senegal

**DOI:** 10.3390/ijerph23030320

**Published:** 2026-03-04

**Authors:** Seynabou Coundoul, Nouhou Diaby, Sophie Déli Tène, Sarbanding Sané, Mohamed Souaré, Auriza Sophia Sylla, Modou Dieng, Lorelay Mendoza Grijalva, Becaye Sidy Diop, Papa Samba Diop, Samba Cor Sarr, Habsatou Tall, Seydou Niang, William Abraham Tarpeh, Abou Abdallah Malick Diouara

**Affiliations:** 1 Laboratoire de Traitement des Eaux Usées (LATEU), Institut Fondamental d’Afrique Noire (IFAN), Cheikh Anta DIOP University, 33 Rte de la Corniche Ouest Dakar, Dakar 10700, Senegal; seynaboucoundoul@esp.sn (S.C.); mohamedsouare@esp.sn (M.S.); aurizasophiasylla@esp.sn (A.S.S.); habsatoutall@yahoo.fr (H.T.); seydou.niang@ucad.edu.sn (S.N.); 2 Groupe de Recherche Biotechnologies Appliquées & Bioprocédés Environnementaux (GRBA-BE), Laboratoire Eau—Énergie—Environnement—Procédés Industriels (LE3PI), Ecole Supérieure Polytechnique (ESP), Cheikh Anta DIOP University, B.P 5085 Dakar-Fann, Dakar 10700, Senegal; sophiedelitene@esp.sn (S.D.T.); sarbandingsane@esp.sn (S.S.); 3 Laboratoire d’Analyses et Essais (LAE), Laboratoire Eau—Énergie—Environnement—Procédés Industriels (LE3PI), Ecole Supérieure Polytechnique (ESP), Cheikh Anta DIOP University (UCAD), B.P 5085 Dakar-Fann, Dakar 10700, Senegal; modou.dieng@ucad.edu.sn; 4 Department of Civil and Environmental Engineering, Stanford University, Stanford, CA 94305, USA; lorelay@stanford.edu (L.M.G.); wtarpeh@stanford.edu (W.A.T.); 5 DELVIC Sanitation Initiatives, Dakar 15542, Senegal; becaye.diop@delvic-si.com; 6 National Sanitation Office, Dakar 11500, Senegal; papsambadiop@gmail.com; 7 Ministry of Health and Social Action, Dakar 10700, Senegal; sambacor.sarr@eradsn.org

**Keywords:** environmental surveillance, wastewater, enteric pathogens, emerging diseases

## Abstract

**Highlights:**

**Public health relevance—How does this work relate to a public health issue?**

- Wastewater-Based Epidemiology (WBE) may serve as a complementary tool to clinical surveillance within the Senegalese context.
- WBE facilitates a more comprehensive understanding of pathogen circulation at the community level.

**Public health significance—Why is this work of significance to public health?**

- The findings demonstrate the feasibility and significance of implementing wastewater-based epidemiology (WBE) in Senegal.
- Adoption of this approach would enable surveillance of enteropathogens and other emerging and re-emerging pathogens.

**Public health implications—What are the key implications or messages for practitioners, policy makers and/or researchers in public health?**

- Wastewater samples contain a large number of pathogens of interest and may influenced by seasonal variations.
- Implementing a WBE program presents an opportunity to strengthen public health strategies, but requires careful consideration of organizational, logistical, and technological factors for effective execution.

**Abstract:**

Despite the importance of wastewater environmental monitoring in disease prevention and response strategies, its use remains poorly documented in Senegal. In addition, there is more onsite sanitation than sewer networks in Dakar, and open drains channel for rainwater are also used as clandestine wastewater discharge into the sea. This study aimed to assess the presence of specific pathogens in wastewater, faecal sludge, and bathing water (the sea). Samples were taken at treatment plants, an open drain, and in the receiving environment (the sea) from June to December 2023. Total nucleic acid was subjected to multiplex qualitative qPCR using SeeGene Allplex™ kits targeting 34 gastrointestinal pathogens. Descriptive statistics, multiple correspondence analysis (MCA) and logistic regression were performed. Considering all matrices, across 51 analysed samples, the results revealed strong bacterial (96.08%, n = 49), parasitic (84.31%, n = 43), and viral (68.63%, n = 35) presence. These results showed high levels of *Aeromonas* spp. (96.08%), *Blastocystis hominis* (80.39%), Enterocytozoon (58.82%), and Norovirus GII (74.51%) among bacteria, protozoa, helminths, and viruses, respectively. Moreover, faecal sludge and pumping station samples show more identified pathogen than wastewater treatment plant and seawater samples. The MCA revealed that the dry season is spatially associated with a greater number of pathogens than the rainy season, but the latter showed a greater species diversity. Logistic regression showed that certain physicochemical parameters, including BOD5, turbidity, pH, and suspended solids, influence pathogen detection. However, qualitative detection and sampling period may constitute limitations. These results reveal that wastewater and bathing water can serve as sources of information on the circulation of pathogens of interest with epidemic potential. Therefore, this valuable epidemiological tool could serve as an adjunct to clinical surveillance in order to prevent future epidemics.

## 1. Introduction

Wastewater is one of the main sources of transmission of certain waterborne diseases, which are associated with several pathogens, including bacteria, parasites, and viruses [1]. Unfortunately, these pathogens can persist in the environment for weeks or months and are challenging to remove with conventional wastewater treatment processes [2,3,4]. They can be released into the environment from municipal wastewater systems and contaminate the food chain through public water supply systems, which may directly or indirectly [5]. The persistence of these pathogens can be influenced by physicochemical factors, such as temperature and pH, among others, which can provide favourable conditions for their survival.

Whether treated or not, wastewater is discharged into the environment, potentially carrying pathogens. Hence, they need to be monitored to prevent the spread of agents responsible for disease burden. Wastewater contains information on pathogen spread, evolution, and outbreak risk [6] and has been successfully used in different contexts [7]. Firstly, environmental monitoring through wastewater surveillance has been extensively used to track the re-emergence of poliovirus in several countries [8]. However, its use increased during the COVID-19 pandemic [7]. Studies carried out in America have shown that virus detections in wastewater preceded the incidence of new COVID-19 cases in the population, and the quantity of viral RNA in wastewater correlated with the cumulative number of cases estimated in clinics [9,10]. This information could help to assess and prevent an epidemic two to three weeks before clinical cases occur, enabling public health authorities to refine their decision-making, especially in resource-limited settings [11]. Also, sewage surveillance has been studied for other enteric viruses and antimicrobial resistance [11,12]. Despite the established value of wastewater surveillance for monitoring poliovirus and its emerging importance during the COVID-19 pandemic, most global public health surveillance systems (including in Senegal) still depend heavily on data from medical cases [13]. However, these clinical data do not take into account people who are not in contact with the health system and may transmit infections, and therefore do not accurately reflect contamination at the population level. Consequently, wastewater-based epidemiology is now a valuable tool for environmental pathogen monitoring, helping reduce the occurrence of certain emerging diseases [14]. This is especially important, as studies confirm that the number of pathogens in wastewater can reflect contamination levels in the community [15].

It has been shown that faecal indicator bacteria (FIB) concentrations do not reflect protozoa and viral pathogens, which pose a real threat of disease, and therefore do not always allow a proper assessment of the overall microbial risks associated with water resources [16]. Furthermore, microbiology alone only detects viable, cultivable organisms [17]. This highlights the need for new approaches, such as molecular methods, to enable rapid detection of challenging target microorganisms in environmental samples. These techniques also represent a promising complementary tool for monitoring microbial indicators to assess water quality and related diseases [18].

In Senegal, wastewater-based epidemiology is not well documented. However, it was shown that respiratory, enteric, and faecal indicators are persistent in faecal sludges [19]. However, except for poliovirus, pathogen environmental surveillance is not integrated into national epidemic surveillance systems. In support of clinical surveillance, this study aims to assess the presence of pathogens known to cause gastrointestinal diseases in wastewater, fecal sludge, and bathing water. This will provide new data that could be used to prevent the emergence and re-emergence of diseases and to prepare for and respond to future epidemics.

## 2. Materials and Methods

### 2.1. Wastewater Sampling Sites

**Figure 1:**
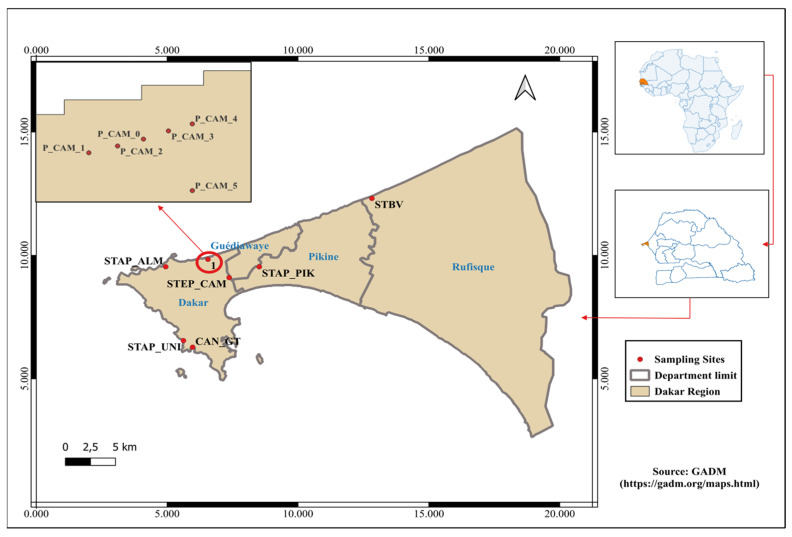
Sampling sites locations. STEP_CAM (Camberene wastewater treatment plant), P_CAM_5 (Wastewater discharge point at Camberene beach), P_CAM_0 (the wastewater/seawater mixing point on the shore at Camberene beach), P_CAM_1 and P_CAM_2 (two sampling points located at 100 to 200 m left from the mixing point), P_CAM_3 and P_CAM_4 (two sampling points located at 100 to 200 m right from the mixing point), STAP_ALM (Almadies pumping station), STAP_UNI (Dakar University pumping station), STAP-PIK (Pikine pumping station), STBV (Tivaouane Peul Fecal Sludge Treatment Plant). Camberene, Almadies, Pikine and Tivaouane Peul are neighborhood in Dakar. Source: GADM (https://gadm.org/maps.html, accessed on 30 November 2025).

This study was carried out in wastewater networks and faecal sludge discharge of Dakar, Senegal, from June to December 2023 (4 sampling campaigns). Seven collection sites were selected for a total of thirteen to fifteen sampling points, depending on sample availability (Figure 1). Specifically, samples were taken from three pumping stations (PS) located at Almadies, Pikine, and Dakar University, a wastewater treatment plant (WWTP) in Camberene, a seawater receiving wastewater discharges (Camberene beach), a faecal sludge treatment plant (FSTP) located in Tivaouane Peul, and an open drain named Canal Gueule Tapée. Sampling points and their main characteristics are described in Table 1.

The pumping stations (PS) are lift stations located in neighborhoods and represent the first wastewater collection stations (from houses) from where wastewater is discharged to the wastewater treatment plants. The wastewater treatment plant (WWTP) receives wastewater from pumping stations for treatment before discharge into the sea. The seawater sampling site is the receiving environment where wastewater, treated or untreated, is discharged (and used by the population as bathing water). A faecal sludge treatment plant (FSTP) is a station where dumping trucks empty sludge collected from onsite sanitation for pre-treatment before discharging it into the wastewater treatment plant. Open Drain (OD) refers to rainwater channels that are illegally used as wastewater channels and discharged into the sea.

The selection of these sites was based on several factors. In the literature, most environmental monitoring studies of wastewater are conducted in sewer systems [19]. Given local realities, this study includes both sewered and non-sewered sanitation systems. There are more non-sewered than sewered sanitation systems in Dakar, covering many more communities. In addition, it has been found that the rainwater open drains are also used as “illegal” wastewater networks. Pumping stations are closest to communities. Analysis of these locations will enable monitoring of trends on a neighborhood-by-neighborhood basis, as well as comparing them with the treatment plants to study possible changes in contamination dynamics.

### 2.2. Sample Collection

Sampling was carried out at the above-mentioned sites during four (04) campaigns: Campaign 1 (June), Campaign 2 (August), Campaign 3 (October), and Campaign 4 (December). Almadies, Pikine, and the Dakar University pumping stations each count as one sampling point. The Camberene wastewater treatment plant has three sampling points: influents (STEP_CAM_ENT), the supernatant obtained after decantation (STEP_CAM_SOR), and effluent after biological treatment (STEP_CAM_BIO). The Camberene beach was divided into six points apart: the wastewater discharge point (P_CAM_5), on the shore at the wastewater/seawater mixing point (P_CAM_0), and two sampling points located at 100 to 200 m left (P_CAM_1 and P_CAM_2) and right (P_CAM_3 and P_CAM_4) from the mixing point. The faecal sludge plant was divided into two points: raw sludge (STBV_BB) and supernatant (STBV_SU), and the open drain had one sampling point at the end of the channel, just before discharge into the sea. Each sampling point was collected in triplicate. Samples were collected using a sterile ladle, then placed in 1-liter sterile bottles, kept cool in a cooler with an ice pack, and transported to IFAN’s wastewater treatment laboratory (LATEU), Dakar University. Samples were taken in the morning between 9 a.m. and 12 p.m., corresponding to the daytime when water use in the concessions is highest and when wastewater flows into the plants are at their peak.

### 2.3. Sample Processing

Pathogens known to cause gastrointestinal illness and that serve as reference microorganisms for national and international legislation and guidelines relating to water reuse were selected [20]. Samples are first deactivated in a Bain-Marie at 60 °C for 60 min to ensure personnel safety. Extraction was performed using the ZymoBIOMICS™ DNA/RNA Miniprep Kit (Zymo Research Corporation, Irvine, CA, USA). In brief, samples (750 µL) were prepared by transferring them into a DNA/RNA Shield™ (Zymo Research Corporation) solution (an equal volume) into a ZR BashingBead Lysis Tube (Zymo Research Corporation). Mechanical homogenization was performed to achieve complete lysis by securing the samples in a high-speed bead beater (Disruptor Genie, Scientific Industries, Inc., Bohemia, NY, USA). Samples were centrifuged, and the supernatant was directly used to extract total nucleic acid (TNA). After extraction, each triplicate was pooled to form a single sample per point and campaign, i.e., 13 or 15 samples per campaign, depending on station availability.

To assess the potential presence of certain pathogenic microorganisms, samples were subjected to an absence/presence multiplex real-time PCR using the SeeGene Allplex Gastrointestinal Panel Assays on a Bio-Rad CFX 96 thermalcycler (Bio-Rad Laboratories Inc., Hercules, CA, USA) according to the manufacturer’s instructions. The panel consists of four kits: the Allplex™ GI-Bacteria (I), Allplex™ GI-Bacteria (II) Assay, Allplex™ GI-Virus Assay, and Allplex™ GI-Parasite Assay kits. These kits are a rapid multiplex polymerase chain reaction platform that can simultaneously detect bacterial, viral, and protozoal agents. The Allplex™ GI-Helminth(I) Assay kit has also been used for helminth detection. Internal control is integrated into the product as an exogenous control of the entire process to verify possible PCR inhibition and co-amplified with target nucleic acids. Amplification was performed in a 25 µL reaction mixture containing 5 µL of nucleic acids extracted from each sample. These kits are dedicated to the qualitative detection of enteric pathogens in human stool specimens (Table 2). The presence of a specific pathogen sequence in the reaction is reported as a Ct value. According to the manufactured information, a Ct limit value is considered positive for the targeted pathogens (Ct ≤ 40 for viruses, Ct ≤ 43 for parasites, and Ct ≤ 45 for bacteria). Data interpretation was performed using the Seegene Viewer software version 3.24.000, which automates result analysis for each target in a valid run and reports whether each target is detected or not.

For the physical and chemical analyses, several pollution parameters were assessed. Electrical conductivity (CE), hydrogen potential (pH), temperature (Temp), turbidity (Turb) and dissolved oxygen (O2), were evaluated in situ using electrochemical methods with HI9813-61 pH/EC/TDS/Temperature Meter (HANNA Instruments, Woonsocket, RI, USA), the Portable Turbidity Meter Turb^®^ 355 IR (WTW, London, UK) and the HQ30D Portable Dissolved Oxygen Meter (HACH, Loveland, CO, USA), respectively. Suspended solids (MES) were determined gravimetrically using a 0.45 µm membrane, followed by heating at 105 °C for 1 h. Chemical oxygen demand (COD) was determined using a DR6000 UV-VIS (HACH), and BOD5 was measured using an Oxitop BOD meter (WTW).

### 2.4. Statistical Analysis

Descriptive analyses were carried out using Microsoft excel^®^ 2016. To determine the relationship between campaigns, sites, and species, multiple correspondence analysis (MCA) was carried out in RStudio version 4.0.1 using the FactomineR package and the factoshiny interface. Sites and campaign factors were treated as qualitative supplementary variables, and the Shapiro–Wilk test was used to assess their influence on the data. To evaluate the association between pathogen detection and physicochemical parameters, logistic regression was performed. A regression model was produced with generalized linear models (GLMs) for each pathogen, including all available physicochemical parameters. Continuous parameters were normalized using a Z-score transformation to facilitate comparison of effects between parameters with different scales. Associations yielding infinite odds ratios were excluded from the analysis. Significant associations were further verified by bootstrap resampling to estimate their stability, followed by Bonferroni and Holm corrections. Extreme odds ratios (>100) were capped at these limits to preserve information and ensure analytical robustness. Odds ratios and their 95% confidence intervals were calculated. All statistical analyses were performed with a significance threshold set at *p* ≤ 0.05.

## 3. Results

### 3.1. Descriptive Statistics

**Figure 2:**
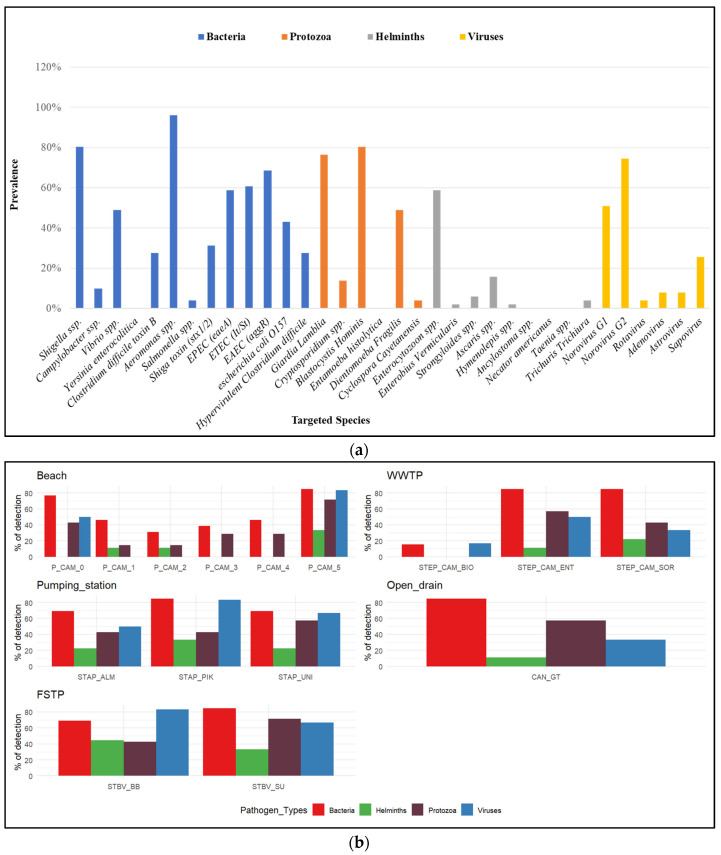
Pathogens detection in samples: (**a**) Pathogens frequency according to samples; (**b**) Distribution of pathogens among sample type. The x-axis corresponds to the sampling sites. WWTP: wastewater treatment plant; FSTP: faecal sludge treatment plant.

A total of 4 sampling campaigns were conducted to screen for 34 gastrointestinal pathogens. Of 51 samples analysed from all sites, the results reveal a strong predominance of bacteria among samples (96.08%, n = 49). Parasites and viruses are present in 84.31% (n = 43) and 68.63% (n = 35), respectively. Regarding the 34 targeted pathogens, 85.29% (n = 29) were detected in the following proportions: bacteria (12/13), viruses (6/6), and parasites (11/15). Detailed bacterial analysis shows that, out of all samples collected, *Aeromonas* spp. and *Shigella* spp./EIEC species were the most frequently identified at 96.08% and 80.39%, respectively. This trend is followed by *E. coli* pathovars, with Enteroaggregative *E. coli* (68.63%), Enterotoxigenic *E. coli* (60.78%), and Enteropathogenic *E. coli* (58.82%) being the most common. *Campylobacter* spp. and *Salmonella* spp. were the least detected, with 9.8% and 3.92%, respectively. All targeted viruses were detected, with Norovirus GII (74.51%) and Norovirus GI (50.98%) being the main representatives. Other viruses were also identified, including Sapovirus (25.49%), Astrovirus and Adenovirus (7.84% in both cases), and Rotavirus (3.92). The most predominant protozoa were *Blastocystis hominis* (80.39%), *Giardia lamblia* at 76.47%, and *Dientamoeba fragilis* (49.02%). Helminths are represented by *Enterocytozoon* spp. (58.82%) and *Ascaris* spp. (15.69%), among others (Figure 2a).

The comparison between samples showed a higher detection rate of enteric pathogens in faecal sludge and pumping-stations samples, followed by WWTP and seawater samples. Regarding the evaluation between points of each site, faecal sludge shows differences in the pathogen detection with increased bacteria and parasites after decantation, ranging from 69.2% to 84.6% for bacteria and from 42.9% to 71.4% for protozoa. On the other hand, we note a reduction in helminths and viruses, from 44.4% and 83.3% to 33.3% and 66.7%, respectively. For pumping stations, Pikine showed higher rates than Almadies and the University sampling sites for all pathogen types. The Camberene WWTP also reveals a remarkable reduction in pathogens as the treatment stages progress. Samples at the inlet show detection rates of 84.6%, 57.1%, 11.1%, and 50.4%, respectively, for bacteria, parasites, helminths, and viruses; this is followed by the decantation outlet (84.6%, 42.9%, 22.2%, and 33.3%) and the outlet after biological treatment (15.4%, 0%, 0%, and 16.7%). The open-air channel showed the same trends as the Camberene station inlet. Samples collected on the beach showed fewer pathogen detections and distinct distributions at different collection points. The wastewater/seawater interface (P_CAM_0) showed a very high pathogens concentration compared with samples collected from the left and right of this point (Figure 2b).

In positive samples, both mixed pathogen co-detection (across different pathogen groups) and intercategory co-detection (within the same pathogen group) were observed. Triplet combinations were analyzed among pathogens within the same group: bacteria (170), viruses (11), protozoa (10), and helminths (2). The most frequently observed triplet combinations and their occurrences in the samples are presented in Table 3.

The physicochemical results were compared with standard data established by local authorities and the World Health Organization on effluent discharge guidelines. The results obtained indicate that, except for temperature (22.3 to 40.4), pH (6.92 to 8), dissolved oxygen (0.11 to 9.7 mg·L^−1^) and turbidity (0.53 to 541.2 NTU), the other pollution indicators, namely suspended solids (23.67 to 4240 mg·L^−1^), BOD5 (10.55 to 8264.33 mg·L^−1^), COD (565.4 to 135.33) and electrical conductivity (709.8 to 53530 µS/cm) exceed the permissible limits prescribed by standard norms.

### 3.2. Multiple Correspondence Analysis

**Figure 3:**
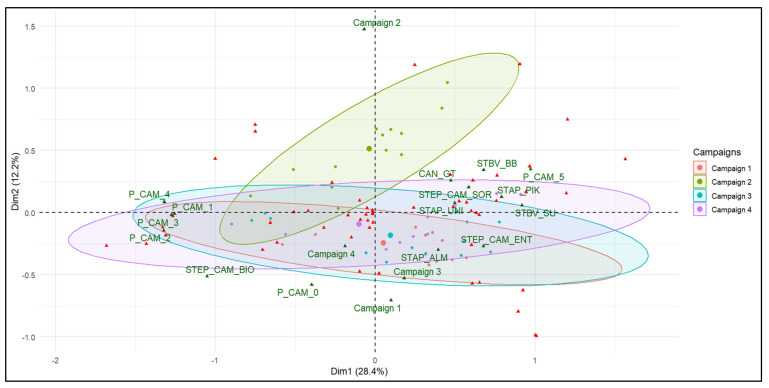
Multiple correspondence analysis (MCA) according to campaigns.

The multiple correspondence analysis (MCA) on the data shows that the first two axes explain 40.6% of the dataset’s variability, with proportions of 28.4% for axis 1 and 12.2% for axis 2 (Figure 3). The critical probability of Wilk’s test indicates that “campaigns” (time) is the factor that best explains the individual variability (*p* = 7.841692 × 10^−14^). The campaigns #1, #3, and #4, held in June, October, and December, respectively, show similar characteristics, indicating that the same species were found in these campaigns (dry season). In contrast, campaign 2 (August, rainy season) presents a distinct profile with a greater diversity of observed individuals, characterized by the presence of particular species such as *Salmonella* (Sal), Hypervirulent *Clostridium difficile* (CD_hyper), *Trichuris Trichiura* (TT), Ascaris (AS), astroviruses (ASV), and shiga toxin-producing *E. coli* (STEC). Concerning the different sample types used, dimension 1 contrasts two clusters: STEP_CAM_ENT, STEP_CAM_SOR, STBV_BB, STBV_SU, STAP_ALM, STAP_PIK, STAP_UNI and CAN_GT), corresponding to samples from sewage and faecal sludge samples on the one hand, and seawater samples (P_CAM1, P_CAM2, P_CAM3, and P_CAM4) and those obtained after biological treatment on the other (Figure 3). Dimension 2, meanwhile, shows slight differences between sludge from pumping stations, treatment plants, and seawater samples. Indeed, we note a difference between samples taken 100 m and 200 m from the seawater/wastewater interface.

### 3.3. Regression Analysis Fundings

**Figure 4:**
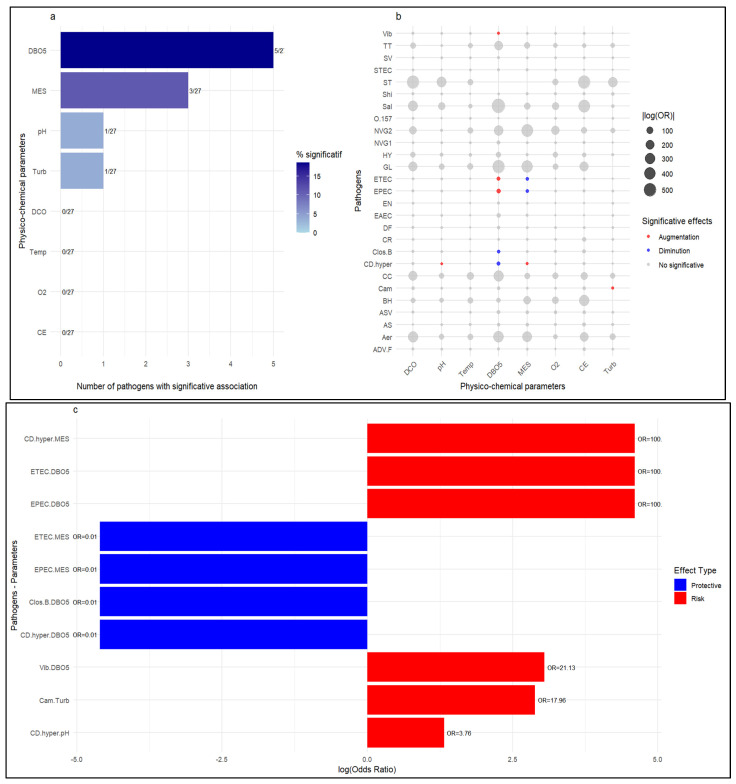
Environmental factors’ influence on pathogen detection: (**a**) Number of pathogens with significative association; (**b**) Effects of physicochemical parameters on pathogens; (**c**) Odds ratio by parameter–pathogen association.

Logistic regression indicated that several physicochemical parameters significantly influenced pathogen detection. Of the 213 associations tested, 4.7% (n = 10) show significant associations with odds ratios ranging from 0.01 to 100 after normalization. These included BOD5 (associated with 5/27 detected pathogens), suspended solids (3/27), pH, and turbidity (1/27, respectively) (Figure 4a). These parameters either influence the growth of pathogens, increasing the likelihood of detecting the pathogens associated with them, or inhibit growth, reducing in the likelihood of detection. However, temperature, conductivity, dissolved oxygen, and COD are not associated with the detection of any pathogens. The results in Figure 4b,c show that an increase in BOD5 multiplies the chances of detecting species such as Vibrio (OR = 21.13) and certain *E. coli* pathovars (ETEC and EPEC, OR > 100). Turbidity increases the chances of detecting *Campylobacter* (OR = 17.96). Similarly, the detection of hypervirulent *Clostridium difficile* is strongly associated with pH (OR = 3.76) and suspended solids (OR = 1.01).

## 4. Discussion

This study aimed to utilize the WBE surveillance approach to assess the presence of enteric pathogens in wastewater, faecal sludge, and bathing water (i.e., seawater). To the best of our knowledge, this was a pioneering study in Senegal that used multiplex real-time PCR to detect pathogens in environmental matrix samples. These tests are designed for human stool samples, but are relatively well-suited to wastewater. They simultaneously detected a large number of bacterial, viral, and parasitic pathogens using nucleic acid testing. Compared with conventional stool tests, these panels offer several advantages, including reduced technical expertise requirement, shorter turnaround times, a consolidated workflow, and increased sensitivity [21].

### 4.1. Pathogen Detection

We identified pathogens in 96.08% of the samples tested. Overall, bacterial detection exceeds viral and parasitic detection. *Aeromonas* was detected significantly among bacteria. Kim et al. found this species in 21.2% of other bacterial species in WWTPs in South Korea [22]. A significant observation was the greatest detection of EAEC, defined by the presence of aggR, and ETEC, defined by the presence of it/st, in most positive samples. These results are similar to those obtained by Ahmad et al. The only difference was the detection of EPEC, defined as the detection of the intimin gene eaeA but at a lower rate [21].

Despite lower Adenovirus detection than in earlier studies [23], Norovirus GI and GII were the most common viruses identified. In contrast with other studies, rotaviruses are reported to have the highest prevalence in wastewater [24,25,26]. This viral dominance by noroviruses is in line with study Koo et al. which suggest that noroviruses have been the leading cause of viral gastroenteritis epidemics worldwide since the introduction of rotavirus vaccines [27]. These pathogens cause high mortality depending on a country’s level of development [28]. Hall et al. consider Norovirus as the leading cause of single-etiology outbreaks in the United States [29]. Sapovirus and Astrovirus are known to cause gastroenteritis in adults and children [30,31]. *Blastocyctis hominis* was the most commonly detected parasite. This pathogen was detected in 80.39% of samples, a rate similar to that reported by Hachich et al. in Brazil [32]. Our results also reveal codetections with *Giardia lamblia*, *Blastocystis hominis*, and *Dientamoeba fragilis* in most of the samples. Bayo et al. found the occurrence of these species in stool samples from children [33].

### 4.2. Seasonality and Spatial Detection

Pathogen detection by season seem to show a higher detection during the dry season, corresponding to sampling campaigns #1, #2 and #3. However, there is a greater species diversity in samples collected during the rainy season (August). These differences are mainly due to several new species that were more abundant in samples collected during the rainy season. Several studies indicate that specific species are mobilized during wastewater flooding, potentially increasing risk to public health [34,35].

According to sample type, those from pumping stations and faecal sludge treatment plants showed a higher number of detected pathogens than those from WWTPs. This difference could be explained by proximity to habitats. Indeed, the pumping stations and faecal sludge treatment plants receive effluent directly from houses, whereas effluent arriving at WWTPs passes through sewer systems and is therefore diluted. This highlights the difference in variability and stability among the matrices. Sewage sludge is recovered from several types of on-site sanitation systems, where it can remain for a very long time, depending on the frequency of emptying, and therefore varies greatly in consistency.

In contrast, wastewater is transported directly from homes to the treatment plant via a gravity-fed network. It is mixed during transport, making it more homogeneous. These faecal sludge matrices may contain waste of any size and type, making it a significant source of infectious pathogens [19]. Moreover, samples collected on the beach showed fewer pathogens, with higher detection at the seawater/sewage interface. These results may be primarily attributed to seawater dilution and environmental conditions [36,37]. For example, the pH and salinity of seawater may affect these microorganisms [38]. However, this contamination of the receiving environment should not be neglected. It may affect bathing water quality and, in the long term, on living matter and sediments [39].

### 4.3. Logistic Regression Findings

Several environmental parameters also influence pathogen detection. BOD5 has the most significant influence on pathogen detection, affecting 5/27 pathogens. A high BOD indicates a significant load of biodegradable organic matter, which serves as a nutrient source and supports the growth of microorganisms [40]. This may explain its strong association with the presence of specific pathogens. However, hypervirulent *Clostridium difficile* is the most sensitive pathogen to parameters (MES, pH, and BOD5). This species is known to be opportunistic, and studies have shown that it is susceptible to environmental pH, with viable spore production being reduced at low pH [41]. It was frequently detected during campaign #2, with neutral to slightly alkaline pH levels. This further promotes their presence and eventual detection. The same applies to suspended solids, which can adsorb these pathogens, thereby reducing their detection. However, some pathogens remain unaffected by environmental pressures and can survive in varying water conditions [42].

### 4.4. Public Health Implications

The detection of pathogens in wastewater can serve as an indicator of infection cases among the population served by the various collection sites. The diversity of pathogens in wastewater has been shown to reflect the pattern of infection in the human population [43]. These findings could further encourage public health authorities to implement an environmental wastewater monitoring program enabling the establishment of an early warning system for the emergence of epidemics.

### 4.5. Limitations

This study adds value to the wastewater detection method in this Senegalese context. Indeed, in addition to the low percentage of cultivable organisms, conventional methods are subject to time constraints that can delay pathogens detection and decision-making. Therefore, PCR amplification is presented as an alternative for expanding the range of indicator organisms used to better specify the causative agents in epidemics, in addition to its rapid detection. Furthermore, using an amplification kit designed for stool specimens in wastewater could underestimate the true prevalence of pathogens, introducing a bias in pathogen detection. This study was conducted between June and December 2023, which does not take into account all seasonal dynamics. In addition, our qualitative results (presence/absence) do not indicate the pathogen load present in the positive samples. This means that, in the near future, quantification will be necessary to determine the level of contamination and may provide comprehensive information not only about its origin but also about the public health measures required.

## 5. Conclusions

This study combined the detection of several pathogens (viruses, bacteria, and parasites) for the first time in Senegal. The main objective was to use environmental surveillance to assess the presence of a range of pathogens in wastewater and bathing water near the outfall. Our analysis indicates that this pilot study assessed the detection of pathogens in wastewater discharge into the environment. Their detection in wastewater demonstrates the effectiveness of environmental monitoring as a tool that can provide additional data and contribute to community level epidemic surveillance. Based on these preliminary results, extending the sampling period to a full year is expected to yield a more comprehensive understanding of seasonal dynamics in pathogen detection. In addition, viral load or temporal variation in the concentration of these pathogens cannot be estimated due to the use of presence/absence analysis. For pathogen detection, PCR was performed with a kit originally designed for human feces, which may reduce detection sensitivity, especially for pathogens present at lower quantities in wastewater. Nonetheless, our study demonstrates the advantages of this approach as a straightforward, effective means of determining trends in pathogen circulation at the community level. Altogether, these results show the need to evaluate the quantities of detected pathogens and to extend the study to other bathing sites near wastewater outfalls. This will enable sufficient data to be obtained to perform a Quantitative Microbial Risk Assessment (QMRA) and establish quantitative criteria for pathogens, and assess the potential public health risks.

## Figures and Tables

**Table 1:** Characteristic data of samples according to the collection sites.

Sites	Samples	Origins	Sample ID	Geographical Coordinates
Camberene beach	Sea-water	Seawater/wastewater inter-face	P_CAM_0	14°45′57.78″ N, 17°26′50.38″ W
Camberene beach	Sea-water	200 m left of meeting point	P_CAM_1	14°45′56.86″ N, 17°26′53.91″ W
Camberene beach	Sea-water	100 m left of meeting point	P_CAM_2	14°45′57.31″ N, 17°26′52.06″ W
Camberene beach	Sea-water	200 m right of meeting point	P_CAM_3	14°45′58.34″ N, 17°26′48.77″ W
Camberene beach	Sea-water	100 m right of meeting point	P_CAM_4	14°45′58.78″ N, 17°26′50.33″ W
Camberene beach	Wastewater	Wastewater Outfall	P_CAM_5	14°45′54.26″ N, 17°26′47.23″ W
Camberene	Wastewater	Wastewater Treatment Plant (WWTP)	STEP_CAM	14°44′49.57″ N, 17°25′36.48″ W
Pikine	Wastewater	Pumping Stations (PS)	STAP_PIK	14°45′27.66″ N, 17°23′56.02″ W
Almadies	Wastewater	Pumping Stations (PS)	STAP_ALM	14°45′27.96″ N, 17°29′08.06″ W
Fann-Point E-Amitié	Wastewater	Pumping Stations (PS)	STAP_UNI	14°41′06.82″ N, 17°28′09.08″ W
Gueule Tapée	Wastewater	Open Drain (OD)	CAN_GT	14°40′43.59″ N, 17°27′38.34″ W
Tivaouane Peulh	Sewage Sludge	Fecal Sludge Station (FSTP)	STBV_BB	14°49′29.70″ N, 17°17′40.46″ W
Tivaouane Peulh	Sewage Sludge	Fecal Sludge Station (FSTP)	STBV_SU	14°49′29.70″ N, 17°17′40.46″ W

**Table 2:** List of pathogens targeted by multiplex PCR tests.

Bacteria	Protozoa	Helminths	Viruses
*Campylobacter* spp.	*Blastocyctis homonis*	*Ascaris* spp.	*Norovirus GI*
*Aeromonas* spp.	*Giardia lamblia*	*Taenia* spp.	*Norovirus GII*
*Salmonella* spp.	*Cyclospora cayetanensis*	*Strongyloides* spp.	*Sapovirus*
*Hypervirulent clostridium difficile*	*Dientamoeba fragilis*	*Necator americanus*	*Rotavirus*
*Shigella* spp.*/EIEC (Sh/EI)*	*Entamoeba histolytica*	*Hymenolepis* spp.	*Astrovirus*
*Vibrio* spp.	*Cryptosporidium* spp.	*Enterocytozoon* spp.	*Adenovirus*
*Clostridium difficile toxin B*	*-*	*Trichuris trichiura*	*-*
*Enterotoxigenic E. coli (ETEC)*	*-*	*Enterobius vermicularis*	*-*
*Enteroaggregative E. coli (EAEC)*	*-*	*Ancylostoma* spp.	*-*
*Enteropathogenic E. coli (EPEC)*	*-*	*-*	*-*
*Shiga toxin-producing E. coli (STEC)*	*-*	*-*	*-*
*Escherichia coli O157*	*-*	*-*	*-*
*Yersinia enterocolitica*	*-*	*-*	*-*

**Table 3:** Triplets of pathogens most frequent in co-detections.

Taxonomic Category	Combination	Number of Samples
Bacteria	Shi + Aer + EAEC	32
Bacteria	Aer + ETEC + EAEC	31
Bacteria	Shi + Aer + EPEC	29
Protozoa	GL + BH + DF	25
Protozoa	GL + CR + BH	7
Protozoa	GL + CR + DF	6
Helminths	EN + AS + TT	2
Helminths	EN + ST + AS	1
Viruses	NVG1 + NVG2 + SV	10
Viruses	NVG1 + NVG2 + ADV.F	4
Viruses	NVG1 + NVG2 + ASV	4

Shi: *Shigella* spp.; Aer: *Aeromonas* spp.; EAEC: Enteroaggregative *E. coli*; ETEC: Enterotoxigenic *E. coli*; EPEC: Enteropathogenic *E. coli*; GL: *Giardia lamblia*; BH: *Blastocystis hominis*; DF: *Dientamoeba fragilis*; CR: *Cryptosporidium* spp.; EN: *Enterocytozoon* spp.; AS: *Ascaris* spp.; TT: *Trichuris Trichiura*; ST: *Strongyloides* spp.; NVG1: Norovirus GI; NVG2: Norovirus GII; SV: Sapovirus; ADV.F: Adenovirus F; ASV: Astrovirus.

## Data Availability

The raw data supporting the conclusions of this article will be made available by the authors on request.

## Abbreviations

The following abbreviations are used in this manuscript:

FIB	Faecal Indicator Bacteria
PS	Pumping Station
WWTP	wastewater treatment plant
FSTP	Faecal Sludge Treatment Plant
OD	Open Drain
LATEU	Wastewater Treatment Laboratory
TNA	Total Nucleic Acid
PCR	Polymerase Chain Reaction
CE	Electrical conductivity
pH	Hydrogen Potential
Temp	Temperature
MES	Suspended Solids
Turb	Turbidity
COD	Chemical Oxygen Demand
BOD	Biochemical Oxygen Demand
MCA	Multiple Correspondence Analysis
GLM	Generalized Linear Models
QMRA	Quantitative Microbial Risk Assessment
